# Insights into the metabolic responses of two contrasting Tibetan hulless barley genotypes under low nitrogen stress

**DOI:** 10.6026/97320630015845

**Published:** 2019-12-31

**Authors:** Zha Sang, Chunbao Yang, Hongjun Yuan, Yulin Wang, Dunzhu Jabu, Qijun Xu

**Affiliations:** 1 State Key Laboratory of Hulless Barley and Yak Germplasm Resources and Genetic Improvement, Lhasa 850002, China; 2 Institute of Agricultural Research, Tibet Academy of Agricultural and Animal Husbandry Sciences, Lhasa 850002, China

**Keywords:** Tibetan hulless barley, low nitrogen stress, metabolome, polyphenols, glycerolipids

## Abstract

Nitrogen (N) is an essential macronutrient for plants. However, excessive use of N fertilizer for cultivation is an environmental hazard. A good adaption to N deficiency is known in the Tibetan hulless barley. Therefore, it is of interest to complete the metabolic analysis on LSZQK which is a low nitrogen (low-N) sensitive genotype and Z0284 that is tolerant to low-N. We identified and quantified 750 diverse metabolites in this analysis. The two genotypes show differences in their basal metabolome under normal N condition. Polyphenols and lipids related metabolites were significantly enriched in Z0284 having a basal role prior to exposure to low-N stress. Analysis of the differentially accumulated metabolites (DAM) induced by low-N explain the genotype-specific responses. Fourteen DAMs showed similar patterns of change between low-N and control conditions in both genotypes. This could be the core low-N responsive metabolites regardless of the tolerance level in hulless barley. We also identified 4 DAMs (serotonin, MAG (18:4) isomer 2, tricin 7-O-feruloylhexoside and gluconic acid) shared by both genotypes displaying opposite patterns of regulation under low-N conditions and may play important roles in low-N tolerance. This report provides a theoretical basis for further understanding of the molecular mechanisms of low-N stress tolerance in hulless barley.

## Background

Tibetan hulless barley (Hordeum vulgare L. var. nudum) is a major staple crop in the Qinghai-Tibetan plateau, cultivated as livestock feed for centuries. Because of the abundance of dietary fibers in hulless barley flour, which can significantly reduce the risk of type II diabetes and some cardiovascular diseases, it is also used as a functional food [1-4]. Qinghai- Tibetan plateau is one of the harshest conditions for agriculture worldwide because of the numerous environmental stresses endured by plants, such as very high altitude, intensive UV radiation, low oxygen pressure, barren, cold, drought and some other abiotic stresses [5]. In order to better adapt to the extreme environmental conditions of this area, hulless barley has evolved strong endogenous resistance systems to resist these stresses [6]. Nitrogen (N) deficiency is a major hurdle for crop yield and quality [7,8]. However, excessive application of N fertilizer in crop production has brought environmental problems [9].Therefore, elucidating the mechanisms underlying N deficiency tolerance is imperative for developing crops tolerant to low-nitrogen (low-N) stress. Compared with common cultivated barley and other crops, Tibetan hulless barley shows generally better adaption to barren, including N deficiency [6,10] and represents a model crop to study low-N tolerance mechanism.

Plants respond to low-N stress through complex alterations in primary and secondary metabolism, protein synthesis, cellular growth processes, expression of regulatory genes, and other cellular pathways [11]. Low-N stress disrupts the multiple metabolic and energy pathways in plants, changing transporter activity and energy supply, eventually causes an imbalance in the uptake and translocation of some essential nutrients, which leads to reduced yield and grain quality [12]. Metabolites represent the ultimate response of biological systems to genetic or environmental changes [13]. Therefore, metabolic profiling could contribute significantly to the study of stress biology in plants [14]. Metabolomics approach has been employed to study various abiotic stresses including salinity, phosphorus, water, sulfur, oxidative and low-N stresses in plants [11,14,15-20]. In hulless barley, metabolic profiling was conducted on salt stress, drought stress, and powdery mildew infection to uncover important pathways related to these stresses [21-23]. However, to our knowledge, there is still no report about the metabolic profiling in Tibetan hulless barley in response to low-N stress. This study aimed at evaluating with mass spectrometry techniques the metabolic changes in leaves of two Tibetan hulless barley genotypes with contrasting responses to low-N stress.

## Materials and Methods:

### Plant sample preparation:

Two Tibetan hulless barley genotypes, including Z0284 and LSZQK were used in this study. Z0284 was identified as a low-N-tolerant genotype while LSZQK is sensitive to low-N conditions based on results from our previous study [24]. Healthy seeds of the two genotypes were sterilized 15 min by 3.5% sodium hypochlorite solution and washed with distilled water. After that, seeds were germinated in an incubator (temperature day/night: 27/25°C; humidity: 60-70%; dark culture). After germination, the seedlings were transplanted into pots containing charcoal: vermiculite=1:1 and cultured in greenhouse with the same temperature, humidity and natural light. When plants have two leaves, they were transferred into a Hoagland nutrient solution of half strength concentration for 1 day to allow them to adapt to the nutrient solution and then transferred to a nutrient solution of normal concentration for 1 week. Normal concentration Hoagland's nutrient solution formula: (Ca(NO3)2•4H2O = 945 mg/L, KNO3 = 607 mg/L, H_12_N_3_O_4_P = 115 mg/L, MgSO47H2O = 493 mg/L, EDTA-Fe = 2.5 ml/L, Trace elements = 5 ml/L, pH = 6.0). Nutrient solution was renewed every 3 days and air pump was used to oxygenate. Thereafter, half seedlings were maintained in a normal concentration Hoagland nutrient solution (N concentration of 4 mM/L, wherein Ca(NO3)2.4H2O and NH4NO3 were used as N source).The other half of the seedlings was cultured in a nutrient solution with half concentration of N as low-N stress treatment (N concentration of 2 mM/L). Two weeks later, the 3 top fully expanded leaf materials in low-N stressed and control treated plants were harvested and immediately frozen in liquid nitrogen for metabolite extraction.

### Extraction of samples metabolites:

The sample preparation, extract analysis, metabolite identification and quantification were conducted as previously fully described by Zhang et al. [25].

### Metabolite identification and quantification:

The sample extracts were analyzed using an LC-ESI-MS/MS system (HPLC, Shim-pack UFLC SHIMADZU CBM30A system, www.shimadzu.com.cn/; MS, Applied Biosystems 6500 Q TRAP, www.appliedbiosystems.com.cn/). The HPLC effluent was alternatively connected to an electrospray ionization (ESI)-triple quadrupole-linear ion trap–MS/MS system (Applied Biosystems 4500 Q TRAP). The analytical conditions were following as Chen et al. [26]. Metabolite identification was based on the MWDB (http://www.metware.cn/), following their standard metabolic operating procedures. Metabolite quantification was carried out using multiple-reaction monitoring (MRM) [26].

### Differential metabolites analysis:

Differentially accumulated metabolites (DAMs) between control and low-N treatments were based on the variable importance in projection (VIP) ≥ 1 and fold change ≥ 2 or fold change ≤ 0.5 [25]. The differential metabolites were analyzed by Kyoto Encyclopedia of Genes and Genomes (KEGG) enrichment [27]. The heatmap was generated using Tbtools [28].

## Results:

### Metabolite profiling under control and low-N conditions in two contrasting hulless barley genotypes:

**Figure 1:**
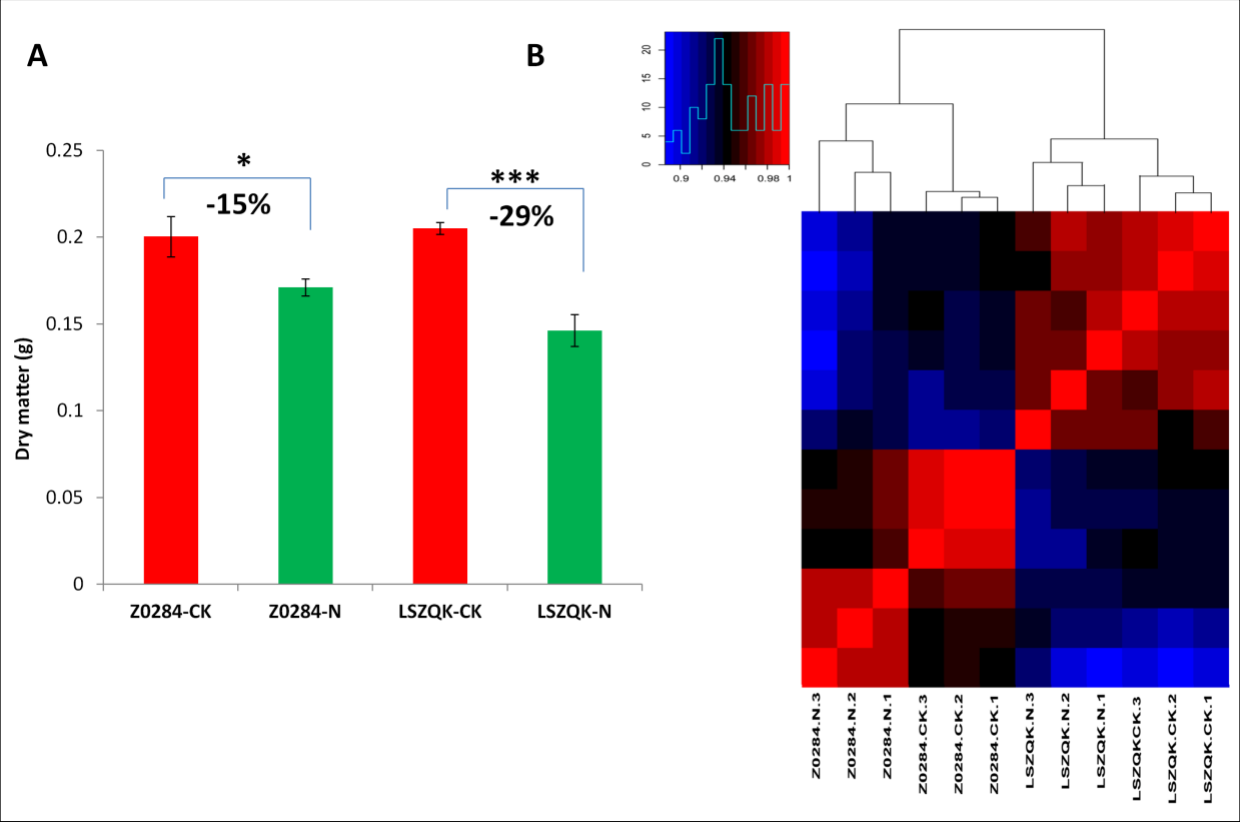
(A) Dry matter (g) of the two genotypes under control and low Nitrogen treatments. (B) Heatmap and hierarchical cluster analysis for detected metabolites in the two genotypes of Tibetan hulless barley under control and low-nitrogen conditions.*, *** means the satistical test was significant at 0.05, 0.001, respectively.

Two genotypes of hulless barley with contrasting responses to low-N (half N concentration as compared to control) treatment were identified after screening a large population (370 varieties) in our previous study [24]. Low-N treatment significantly reduced the dry matter in both genotypes but at a lesser extent (-15%) in Z0284 as compared to LSZQK (-29%) (Figure 1A), indicating that Z0284 is more tolerant to low-N as compared to LSZQK. Ultra performance liquid chromatography - tandem mass spectrometer (UPLC-MS/MS) system was used to profile the metabolites in the leaves of two genotypes under low-N and control conditions.

Totally, 750 metabolites were identified in all samples, which can be classified into 31 types. These metabolites included 71 organic acids, 65 flavone, 63 amino acid derivatives and so on (Table S1). To obtain a global picture of the metabolite profiles in the two genotypes of Tibetan hulless barley in response to low-N stress, all measured metabolite data were subjected to hierarchical clustering analysis (Figure 1B). All the samples from the same genotype were clustered together, showing the reliability of the quantitative data obtained by UPLC-MS/MS. Besides, the two genotypes were divided into two distinct groups regardless of the N treatments, suggesting that N assimilation in these two genotypes is clearly different and affects their metabolic profiles. Control and low-N samples were also clearly separated, showing that low-N stress induced significant changes on the metabolite concentrations in hulless barley.

### Comparison of metabolite contents between Z0284 and LSZQK under control condition: 

From the hierarchical clustering analysis (Figure 1B), we hypothesized that the two genotypes may have differences in their basal metabolome under control condition. Therefore, we compared the metabolite contents in the two genotypes under control condition. The differentially accumulated metabolites (DAM) were selected based on the variable importance in projection (VIP) ≥ 1 and fold change ≥ 2 or fold change ≤ 0.5 [25]. As suspected, we detected 195 DAMs between the two genotypes with 123 up-accumulated and 72 down-accumulated in the tolerant genotype (Table S2). The large number of DAMs and particular the high proportion of up-accumulated compounds in Z0284 clearly demonstrates that this genotype has a distinct metabolic profile from LSZQK, with globally increased contents of polyphenols related metabolites (catechin derivatives, flavanones, flavones, flavone C-glycosides, flavonols, flavonolignans, hydroxycinnamoyl derivatives, isoflavones, etc.) and lipids related metabolites (lipids_fatty acids, lipids_glycerolipids and lipids_glycero phospholipids).

### The changes of metabolite profiles of the two genotypes in response to low-N stress:

**Figure 2:**
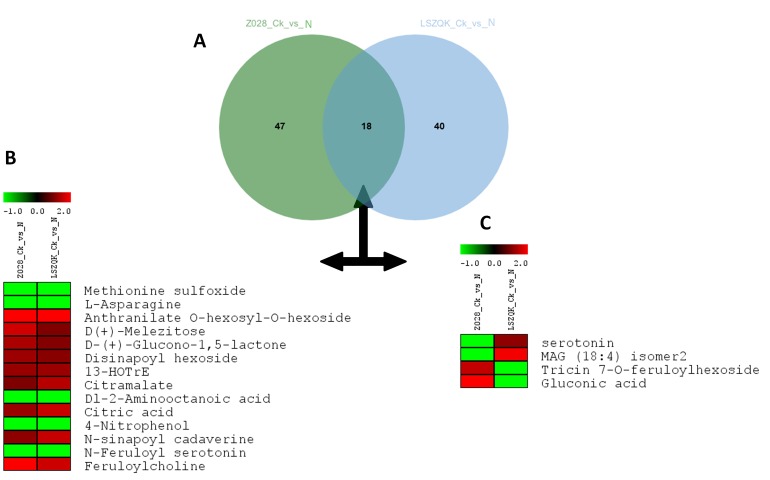
Differentially accumulated metabolites (DAM) in response to low Nitrogen stress in hulless barley. (A) Venn diagram depicting the common and unique DAMs between Z0284 and LSZQK in response to low Nitrogen stress; (B) Heatmap showing the 14 core metabolites with similar concentration changes (log2 fold change) from control condition (Ck) to low Nitrogen condition (N) in Z0284 and LSZQK; (B) Heatmap showing the 4 core metabolites with differential concentration changes (log2 fold change) from Ck to low Nitrogen condition in Z0284 and LSZQK.

In order to investigate the metabolite concentration changes induced by low-N treatment in the two genotypes, a comparative analysis of the metabolite contents between the control and low-N stress conditions was performed for each sample. In total, 58 DAMs (46 up-and 12 down-accumulated) and 65 DAMs (30 up-and 35 down-accumulated) were detected in LSZQK and Z0284, respectively. The proportions of up- and down-accumulated metabolites found within the DAMs of the two genotypes greatly differ, which clearly shows how these two genotypes respond differentially to the low-N stress. Moreover, only 18 DAMs were commonly found in the two genotypes (Figure 2A). Among these, 14 metabolites showed similar patterns of concentration change between low-N and control conditions in the two genotypes, which denotes that these 14 compounds represent the core low-N responsive metabolites regardless of the tolerance level in hulless barley (Figure 2B). Overall, under low-N condition, N-free compounds were increased while N-containing compounds were reduced in their concentrations. The other four DAMs that were commonly shared by the two genotypes (serotonin, MAG (18:4) isomer 2, tricin 7-O-feruloylhexoside and gluconic acid) displayed opposite patterns of regulation under low-N conditions (Figure 2C). These compounds may play important roles in low-N tolerance.

## Discussion:

In the present study, two Tibetan hulless barley with low-N tolerance (Z0284) and low-N sensitivity (LSZQK) were selected from a large population screened in our previous study [24]. In contrast to LSZQK, Z0284 could maintain a high biomass production under limited supply of N (Figure 1A), which denotes that Z0284 is able to keep up a nearly normal metabolism under low-N condition. The content of nearly 1/3 of the global metabolite was significantly different between the two genotypes under control condition, highlighting the intrinsic metabolomic difference between LSZQK and Z0284. Distinctly, polyphenols and lipids related metabolites were significantly enriched in Z0284, which may play a basal role prior to exposure to low-N stress. It has been widely reported that biotic and abiotic factors can induce polyphenol accumulation in plants [29]. Flavonoids are the major stress inducible polyphenols and include flavonols, flavonones, flavones, isoflavones, anthocyanins, etc. [30]. In particular, high levels of flavonoids accumulation induced by low N was found in several plants, including Arabidopsis, Nuphar advena, Potamogeton amplifolius and Cyclocarya paliurus [31-36]. In N-starved tomato plants, an increase of total flavonoids by 14% was noticed [37]. Similarly, cellular lipid levels (mainly glycerolipids) also play essential roles in response to environmental stress [38,39]. In algae, nitrogen depletion has been defined as one of the best lipid accumulator stress condition [40,41]. In Arabidopsis thaliana, lipid biosynthesis was found to be significantly induced by N deprivation [42], indicating that organisms tend to accumulate lipids to combat low-N stress effects. Similarly, the highest of storage lipids in tea plant leaves was found under 0 kg/ha N treatment as compared to 285 and 474 kg/ha N application [43]. Keeping all these in view, we deduce that the high accumulation of lipids and polyphenols metabolites in Z0284 prior to low-N stress condition is advantageous as they can be quickly deployed as key arsenals under low-N stress conditions. Based on this observation, we will further analyze the polyphenol and lipid content under normal growth conditions in more genotypes with contrasting responses to low-N as identified in our previous study [24]. Under low-N stress, we observed that less than 10% of the global metabolome was significantly altered in both genotypes. This proportion is very low as compared to other environmental stresses such as drought [22] and salt [23] in hulless barley, where approximately 75% and 56% of the global metabolome were affected, respectively. It is probable that the relatively tolerance of hulless barley to low-N could be the underlying reason for this weak metabolite change [6,10]. For example, in rice which is a typical low-N sensitive crop, over 80% of the detected metabolites were affected by N starvation [12].

The global metabolic alterations in the two genotypes under low-N stress revealed distinct genotype-dependent metabolite alterations, which is similar to previous reports [10,12,44]. Although individual genotypes show differing abilities to maintain growth and productivity by acclimating to stress conditions through specific tolerance mechanisms [45], there are some conserved mechanisms triggered by all genotypes from the same species or even among different species. Wang et al. [23] reported 13 stress-related metabolites representing the core metabolome in response to salt stress in huless barley. Similarly, Yuan et al. [22] also described 251 metabolites as the core metabolome in response to drought stress regardless of the tolerance level of hulless barley genotypes. In this study, 14 DAMs including, methionine sulfoxide, L-asparagine, anthranilate O-hexosyl-O-hexoside, D(+)-melezitose, D-(+)-glucono-1,5-lactone, disinapoyl hexoside, 13-HOTrE, citramalate, Dl-2-aminooctanoic acid, citric acid, 4-nitrophenol, N-sinapoyl cadaverine, N-feruloyl serotonin and feruloylcholine were commonly identified in both genotypes with the same pattern of accumulation, which indicates that they represent the core metabolome altered in response to low-N stress in Tibetan hulless barley regardless of the genotype or tolerance level. This preliminary result is very important however, it needs to be validated in a large panel. It will help us to pinpoint the most important core low-N responsive metabolites, which could be the targets of in-depth molecular studies aiming at identifying the associated genes that we can manipulate to increase the response of hulless barley to low-N stress. Besides, we also identified four candidate metabolites with different patterns of accumulation between the two studied hulless barley genotypes in response to low-N treatment. Further investigations are needed to uncover the specific roles of these molecules in low-N responses and which strategies could be developed in order to enhance low-N tolerance not only in hulless barley but potentially in other crops as well.

## Conclusions:

In summary, we studied the metabolic response of two contrasting hulless barley genotypes to low Nitrogen (N) stress. Our data suggest that the basal metabolome under optimal N is important for efficient response upon exposure to low N stress. We revealed key classes of metabolites highly active under low N stress independently of the genotype tolerance levels. In addition, the metabolites conferring low N tolerance were also pinpointed. This study generated extensive metabolic data and unveiled key metabolites to target for improving low-N stress tolerance in hulless barley.

## Author Contributions: 

Conceptualization, Q.X., C.Y. and Z.S.; methodology, H.Y.; software, J.D.; validation, C.Y. and Z.S.; formal analysis, Y.W.; investigation, C.Y. and H.Y.; resources, S.Z.; data curation, D.J. writing-original draft preparation, Z.S.; writing, review and editing, Q.X., C.Y. and Z.S.; supervision, Q.X.; funding acquisition, Q.X.
